# Validation of a search strategy for randomized clinical trials related to periodontitis

**DOI:** 10.1186/s13690-021-00560-0

**Published:** 2021-04-03

**Authors:** Amanda Oliveira Lyrio, Simone Seixas da Cruz, Isaac Suzart Gomes-Filho, Viviane Seixas Silva Silveira, Elivan Silva Souza, Josicélia Estrela Tuy Batista, Ana Claudia Morais Godoy Figueiredo, Mauricio Gomes Pereira

**Affiliations:** 1 Faculty of Health Sciences, University of Brasilia, Brasília, Distrito Federal Brazil; 2 Health Sciences Center, Federal University of Recôncavo da Bahia, Rua Fonte do Céu, 1521, Andaiá, Santo Antônio de Jesus, Bahia 44572-560 Brazil; 3 Department of Health, Feira de Santana State University, Feira de Santana, Bahia Brazil; 4 Bibliotheek Schilderswijk, Haia, Holland NL; 5 Faculty of Collective Health, University of Brasilia, Brasília, Distrito Federal Brazil; 6 Epidemiology Surveillance, Federal Distritct Health State Secretariat, Brasília, Distrito Federal Brazil

**Keywords:** Methodological studies, Research design, Systematic review, Periodontitis, Sensitivity

## Abstract

**Background:**

Systematic reviews, considered the gold standard for the assessment of scientific evidence, may present conflicting findings for the same clinical issue, and such dissent may be justified by the forms of elaboration of the electronic search strategy. This paper aims to validate a search strategy to identify randomized clinical trials related to periodontitis. A gold standard reference set was developed to validate the identified clinical trials using the relative recall method. The choice of periodontitis is due to the fact that this disease has a high prevalence among chronic non-communicable diseases, is considered the second most common oral disease in the world, is associated with several health problems, such as cardiovascular diseases and diabetes, and, principally, has not been investigated sufficiently to prevent possible damages resulting from it.

**Methods:**

A validation study was developed in MEDLINE/PubMed. In Stage 1, a methodological filter recommended by the Cochrane Collaboration to identify randomized clinical trials was applied. Stage 2 identified articles related only to periodontitis (gold standard reference set) from among the articles retrieved using the eligibility criteria. In Stage 3, a search statement for the retrieval of periodontitis-related articles was elaborated by experts. Stage 4 defined the proposed search strategy comprising of the combination of the search statement developed with the aforementioned methodological filter and subsequent application in MEDLINE/PubMed. The obtained data were analyzed using the set of articles identified in Stage 2, as the gold standard reference set. The following performance values were calculated - sensitivity, specificity, accuracy, and number needed to read - with their respective 95% confidence interval (95%CI).

**Results:**

The search strategy under evaluation compared to the gold-standard showed a sensitivity of 93.2% (_95%_CI, 83.8–97.3), specificity of 99.9% (_95%_CI 99.8–99.9), and a precision of 77.5% (_95%_CI, 66.48–85.63). In addition, the number needed to read was 1.3.

**Conclusion:**

According to the proposed methodological approach, the search strategy under evaluation performed well in the identification of randomized clinical trials related to periodontitis.

**Supplementary Information:**

The online version contains supplementary material available at 10.1186/s13690-021-00560-0.

## Background

In the last decades, the systematic review, defined as a type of study that synthesizes the scientific evidence existing in the literature, has provided ample space in the field of health, including dentistry. It is estimated that in 2010, 11 systematic reviews were published per day [1]. Most likely, this number is even higher today. Regarding dentistry, it is estimated that approximately 1000 (thousand) systematic reviews were published in 2017, according to the MEDLINE using PubMed platform (MEDLINE /PubMed).

Although it is considered the gold standard for the assessment of scientific evidence, systematic reviews of randomized clinical trials often present conflicting findings for the same issue [2]. Considering the reproducibility of this design, the aforementioned conflict between the findings does not seem justifiable, a priori*.*

However, a closer inspection of the stages of the systematic review protocol may explain this phenomenon. One of them concerns the elaboration of the electronic search strategy, which can be simplified as a “specific algorithm”. The construction of this syntax occurs through index terms/synonyms and symbols to retrieve articles that report evidence about a particular research question in an electronic bibliographic database [3, 4].

However, it should be highlighted that an electronic search strategy holds strong subjectivity in itself, to the point that scholars argue that different researchers invariably tend to build different strategies on the same object of interest [5, 6]. The proper application of filters related to the indexing of the articles, based on the descriptors established, is necessary to minimize this subjectivity of the search. For example, a quick search in the periodontal literature, without proper application of filters, can generate at least 10 different strategies aiming to identify periodontitis-related studies with varied results and that do not truly identify the articles on the topic. The researcher can identify different sets of references retrieved, from 66 to 18,000 sets, with varying accuracy. It can impact directly on the quality of the systematic search and its results as well as the time required for its execution [7–10].

Therefore, strengthening the means to validate search strategies, estimating quantitative indicators of their performance, such as the strategy sensitivity and specificity, is a reasonable way to increase the quality of the identification of studies and, consequently, of the findings of systematic reviews. This article aims to validate a search strategy for the identification of randomized clinical trials related to periodontitis. The choice of periodontitis is due to the fact that the disease is a very important health problem. It also has a high prevalence among chronic non-communicable diseases, is considered the second most common oral disease in the world, is associated with several health problems, such as cardiovascular diseases and diabetes, and, principally, has not been investigated sufficiently to prevent possible damages resulting from it.

## Methods

### Study design and setting

This is a methodological study for the validation of a search strategy to identify randomized clinical trials related to periodontitis on MEDLINE /PubMed. We developed a gold standard reference set to validate the identified clinical trials using the relative recall method. The relative recall indicator (precision) was estimated by dividing the number of gold standard references identified with the search strategy (under validation) by the total of references selected by the proposed search strategy.

### Procedures for identification of the gold-standard set and the retrieved articles using the search strategy under evaluation

#### Stage 1 – application of the Cochrane Collaboration’s methodological filter

Initially, the methodological filter was applied in MEDLINE /PubMed to identify randomized clinical trials, which was validated by the Cochrane Collaboration (Cochrane Highly Sensitive Search Strategy – HSSS) and has high sensitivity and precision for MEDLINE /PubMed [11], as follows:

#1 randomized controlled trial [Publication Type].

#2 controlled clinical trial [Publication Type].

#3 randomized [Title/Abstract].

#4 placebo [Title/Abstract].

#5 clinical trials as topic [MeSH Terms].

#6 randomly [Title/Abstract].

#7 trial [Title].

#8 #1 OR #2 OR #3 OR #4 OR #5 OR #6 OR #7.

#9 animals [mh] NOT humans [mh].

#10 #8 NOT #9.

A chronological filter was also used from January 01 to March 31, 2018.

#### Stage 2 – application of the eligibility criteria

Among the articles retrieved concerning randomized clinical trials, only those related to periodontitis were identified, without restriction of language, sex, nationality, and age of participants. In addition, exclusion criteria comprised of studies involving animal models and reviews of randomized clinical trials. This stage of reading titles and abstracts was performed by two authors (SSC and AOL) and confirmed by a more experienced periodontist (ISGF), in case of disagreement. When necessary, full-text versions were evaluated. Thus, after the identification of all references that were found, using the Cochrane filter and related to Periodontics, this set was considered as the gold standard. That is, the gold standard reference set was composed of randomized clinical trials, related to periodontitis.

#### Stage 3 – definition of the search statement related to periodontitis

The search statement for identifying the condition of interest (periodontitis) was developed using the tool “advanced search” in MEDLINE/PubMed, as follows: 1) controlled vocabulary terms related to periodontitis were identified; 2) a periodontist and general dentist (ISGF and SSC) identified the main keywords and their derivations; 3) the retrieved articles were carefully analyzed, and the terms that were associated with studies not related to periodontitis were discarded, for example the term “gingivitis”; and 4) the procedure was repeated until the strategy was considered adequate, using the *Peer Review of Electronic Search Strategies* (PRESS, Additional file- Appendix A) [4] checklist with the assistance of an experienced librarian (VSSS).

Finally, the following search statement for identifying periodontitis was developed:

#1 “Periodontitis” [Title/Abstract].

#2 “Periodontitis” [MeSH Terms].

#3 “Disease, Periodontal” [Title/Abstract].

#4 “Disease, Periodontal” [MeSH Terms].

#5 “Diseases, Periodontal” [Title/Abstract].

#6 “Diseases, Periodontal” [MeSH Terms].

#7 “Periodontal Disease” [Title/Abstract].

#8 “Periodontal Disease” [MeSH Terms].

#9 “Parodontosis” [Title/Abstract].

#10 “Parodontosis” [MeSH Terms].

#11 “Parodontoses” [Title/Abstract].

#12 “Parodontoses” [MeSH Terms].

#13 “Pyorrhea Alveolaris” [Title/Abstract].

#14 “Pyorrhea Alveolaris” [MeSH Terms].

#15 #1 OR #2 OR #3 OR #4 OR #5 OR #6 OR #7 OR #8 OR #9 OR #10 OR #11 OR #12 OR #13 OR #14.

#### Stage 4 – application of the search strategy under evaluation

Stage 4 comprised the combination of the search statement developed (Stage 3) with the abovementioned methodological filter (Stage 1) and subsequent application in MEDLINE/PubMed for the retrieval of randomized clinical trials related to periodontitis, defined as the search strategy under evaluation.

### Data analysis procedures

The proposed search strategy was evaluated by analyzing the extent to which it retrieved the studies in the gold-standard articles set, and the sensitivity, specificity, and precision of this strategy were calculated according to the following definitions:

The sensitivity indicator was estimated by dividing the number of references retrieved by the proposed search strategy that were contemplated by the gold standard set (true positive) by the total of references selected by the gold standard set (true positive + false negative).

The specificity indicator was estimated by dividing the number of references not recovered by the proposed research strategy and which were not contemplated in the gold standard set (true negative) by the total number of references selected by the Cochrane Highly Sensitive Search Strategy (HSSS)that were also not part of the gold standard set (false positive +true negative).

The precision indicator was estimated by dividing the number of references retrieved by the proposed search strategy that were contemplated by the gold standard set (true positive) by the total of references selected by the proposed search strategy (true positive + false positive). Precision was also identified in this article as relative recall. Number needed to read indicator was estimated as the inverse of precision.

I – $$ \mathrm{Sensitivity}=\frac{\mathrm{True}\kern0.17em \mathrm{positive}}{\mathrm{True}\kern0.17em \mathrm{positive}+\mathrm{False}\kern0.17em \mathrm{negative}} $$
II – $$ \mathrm{Specificity}=\frac{\mathrm{True}\kern0.17em \mathrm{negative}}{\mathrm{False}\kern0.17em \mathrm{positive}+\mathrm{True}\kern0.17em \mathrm{negative}} $$
III – $$ \mathrm{Precision}=\frac{\mathrm{True}\kern0.17em \mathrm{positive}}{\mathrm{True}\kern0.17em \mathrm{positive}+\mathrm{False}\kern0.17em \mathrm{positive}} $$
IV – $$ \mathrm{Number}\kern0.17em \mathrm{needed}\kern0.17em \mathrm{to}\kern0.17em \mathrm{read}=\frac{1}{\mathrm{Precision}} $$

The 95% confidence intervals (_95%_CI) of the strategy performance values were calculated for each estimated measurement.

## Results

**Fig. 1:**
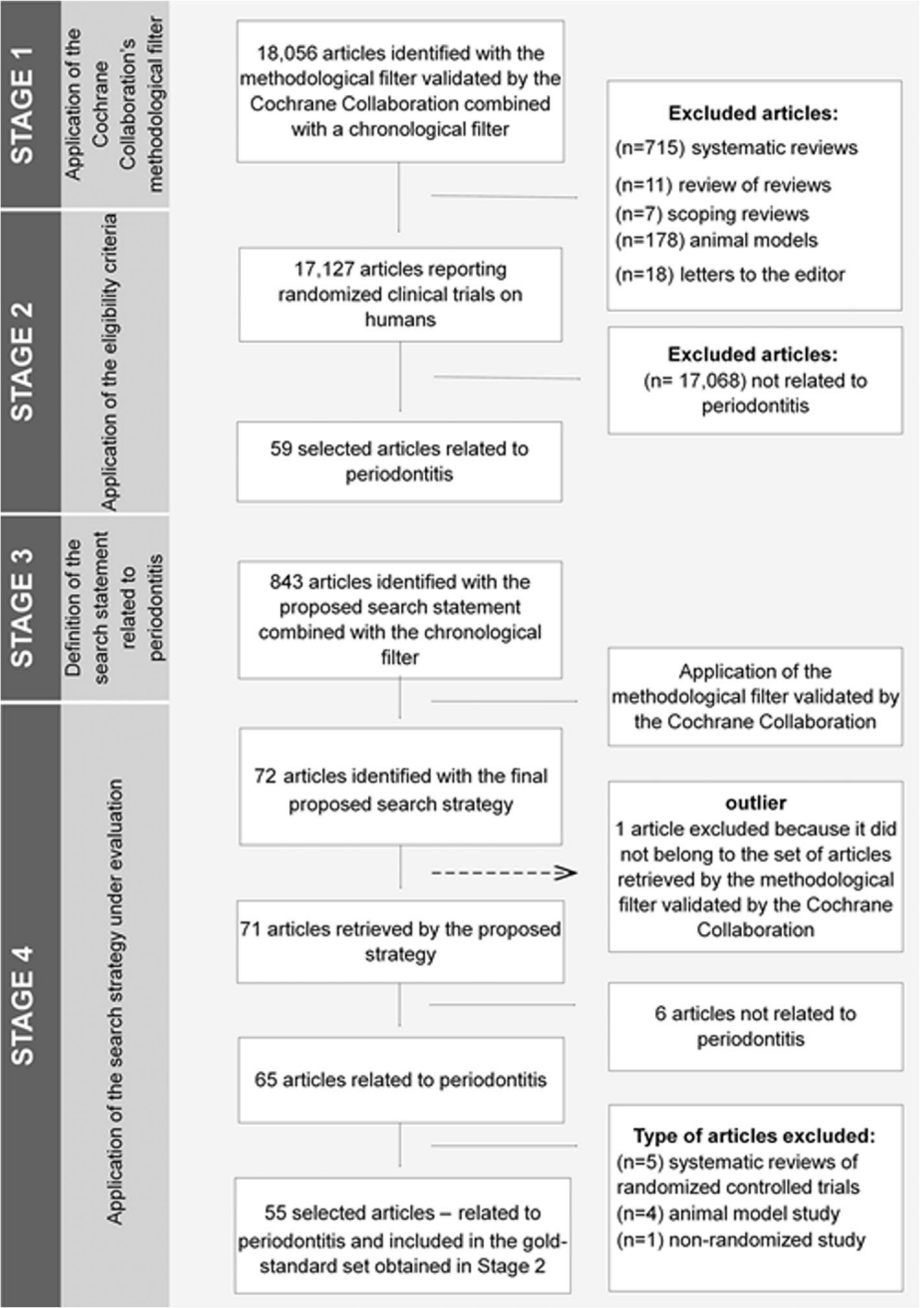
Flowchart of the procedures for identification of the gold-standard set and the articles retrieved using the search strategy under evaluation

At the end of Stage 1, a total of 18,056 articles were retrieved according to the Cochrane Highly Sensitive Search Strategy (HSSS) methodological filter combined with the chronological filter. Of these, 178 were conducted using an animal model, 18 were letters to the editor, seven were scoping reviews, 11 were reviews of reviews, and 715 were systematic reviews of clinical trials (Fig. 1). As for the other studies, 17,127 clinical trials were conducted on humans, of which only 59 were related to periodontitis, comprising of gold-standard articles set obtained in Stage 2.

After the evaluation of the search strategy with PRESS, a search statement was obtained, employing controlled vocabulary terms, title and abstract filters, connected by Boolean operators, combined with a chronological filter. Thus, according to Fig. 1, at the end of the Stage 3, 3843 articles were obtained. By using the final strategy under evaluation, 72 randomized clinical trials related to periodontitis were retrieved at the Stage 4. It is noteworthy, however, that from the total of retrieved articles, one study (1.4%) did not belong to the set of randomized clinical trials identified with the Cochrane Collaboration methodological filter, although this was of interest to the bibliographical search. Therefore, the study was excluded from the analysis, being classified as an outlier. The PubMed search history performed in November, 16th, 2018, can be seen in Additional file- Appendix B.

Among the 71 retrieved articles, 65 were related to periodontitis. However, when only randomized clinical trials were selected, 55 articles were included in the performance analysis of the search strategy under evaluation. The findings of the performance analysis showed that the final search strategy had a sensitivity of 93.2% (_95%_CI 83.8–97.3), specificity of 99.9% (_95%_CI 99.8–99.9), precision of 77.5% (_95%_CI 66.5–85.7) and number needed to read of 1.3 studies (Table 1).

**Table 1:** Sensitivity, specificity, and precision, their respective 95% confidence intervals (_95%_CI), and number needed to read for the comparison between the search strategy under evaluation and the set of gold-standard articles

Indicator	Value	_95%_IC
Sensitivity (%)	93.2	83.8–97.3
Specificity (%)	99.9	99.8–99.9
Precision (%)	77.5	66.5–85.6
NNR * (absolute value)	1.3	–

* Number needed to read (NNR) = 1/ precision

## Discussion

### Main results

The main findings of the present study suggest that the search strategy under evaluation, used to identify randomized clinical trials related to periodontitis in MEDLINE/PubMed, presented a good performance when compared to the gold-standard strategy, based on validity indicators - sensitivity, specificity, precision and number needed to read.

### Comparison with other types of study about the topic

Studies that carried out validation of a strategy to identify randomized clinical trials specifically related to periodontitis were not found. However, there are investigations that validated strategies to identify studies related to other areas. In addition, some studies [11, 12] carried out validation of search strategies employing a method similar to the one presented here, with a gold-standard based also on the HSSS filter of the Cochrane Collaboration.

With a similar goal, a strategy to obtain a sensitive search about randomized clinical trials on diet and nutrition was developed [12]. The gold-standard of the aforementioned study was based on the HSSS, and 298 systematic reviews of the Cochrane Collaboration were employed, rather than original articles as in the present study. Also, it was observed that the best strategy of the study on diet and nutrition showed sensitivity of 88.6%, close to the indicator estimated in this study, 93.2%.

Similarly, in another investigation, an attempt to recognize articles related to adverse effects to surgery [13], the sensitivity of two search strategies was estimated. In their best strategy, sensitivity measurements of 93% for MEDLINE and 95% for Embase were obtained. Thus, again, indicators similar to those estimated in this investigation were also observed.

### Strengths

The search strategy proposed in this study can be well applied to the elaboration of systematic reviews of randomized clinical trials related to periodontitis, since it will promote a reduction in the operational time of an important stage of this type of secondary study - the identification of publications to be included [14–17].

According to one of the performance indicators evaluated, the number needed to read [18, 19], for every 13 articles identified, 10 would likely be of interest to the researchers, conferring a higher operational speed for this stage. Therefore, the elaborated strategy can be useful for reducing time and human resources for the elaboration of bibliographic researches.

Consequently, there can be a considerable cost reduction for the performance of systematic reviews related to periodontitis, which are commonly useful for the synthesis of evidence [5, 7, 20]. In addition, there is an increase in the validity of the review since the strategy developed showed high sensitivity in the identification of studies on the topic of interest.

It should be noted that the adoption of the gold-standard search strategy was based on two pillars. The first one, which has recognized validity, since a filter developed by the Cochrane Collaboration (HSSS) [11] was used to identify all randomized clinical trials in the period determined in this investigation. The second pillar concerns the construction of a search statement, specific for periodontitis, developed independently by two researchers with experience and qualifications in the field of knowledge, thus improving the reliability of the identification of relevant studies.

It is also noteworthy that this search statement was evaluated by a professional with a background in Librarianship, according to the recommendations of PRESS, aiming to improve the quality of the research in the database [21, 22].

### Limitations

The fact that this study only consulted the platform MEDLINE/PubMed can represent a limitation since it restricts the extrapolation of the good performance of the strategy developed to other databases [17]. However, the adaptation of MEDLINE/PubMed search syntax to the other main electronic databases, such as Embase or Web of Science, is a common procedure, which does not require great effort on the part of researchers [5].

Another limitation refers to the chronological filter applied to the Cochrane strategy for the identification of randomized clinical trials, which included the three initial months of the year 2018. This decision provided a convenience sample of the studies published that year, instead of a probabilistic sample that would be more desirable to increase the representativeness of the included studies in the referred year.

In this sense, the next steps for this investigation include the use of all randomized clinical trials over a year to minimize the potential problem of generalized restriction. Another improvement would be an evaluation of the quality of the investigations retrieved using the evaluated strategy, since this step was not performed in this study. It is noteworthy that the proposed search strategy requires the complementation of search by hand, since it is known that this adjuvant resource is important for any high-quality systematic bibliographic search.

## Conclusions

The developed search strategy exhibited good performance for the adequate retrieval of randomized clinical trials related to periodontitis. Additionally, it can be a useful tool in reducing time and cost for researchers.

## Additional file

**Additional file 1 Appendix A.** Peer Review of Electronic Search Strategies (PRESS). **Appendix B.** PubMed search history (11/16/2018).

## Data Availability

The datasets during and/or analysed during the current study available from the corresponding author on reasonable request.

## Abbreviations

HSSS: Highly Sensitive Search Strategy
NNR: Number needed to read

## References

[1] Bastian H, Glasziou P, Chalmers I (2010). Seventy-five trials and eleven systematic reviews a day: how will we ever keep up?. PLoS Med.

[2] Ioannidis JP (2010). Meta-research: the art of getting it wrong. Res Synth Methods.

[3] Sampson M, McGowan J, Cogo E, Grimshaw J, Moher D, Lefebvre C (2009). An evidence-based practice guideline for the peer review of electronic search strategies. J Clin Epidemiol.

[4] McGowan J, Sampson M, Salzwedel DM, Cogo E, Foerster V, Lefebvre C (2016). PRESS peer review of electronic search strategies: 2015 guideline statement. J Clin Epidemiol.

[5] Jenkins M (2004). Evaluation of methodological search filters--a review. Health Inf Libr J.

[6] Franco JVA, Garrote VL, Escobar Liquitay CM, Vietto V (2018). Identification of problems in search strategies in Cochrane reviews. Res Synth Methods.

[7] Faggion CM, Atieh MA, Park S (2013). Search strategies in systematic reviews in periodontology and implant dentistry. J Clin Periodontol.

[8] Natto ZS, Abu Ahmad RH, Alsharif LT (2018). Chronic periodontitis case definitions and confounders in periodontal research: a systematic assessment. Biomed Res Int.

[9] Manrique-Corredor EJ, Orozco-Beltran D, Lopez-Pineda A, Quesada JA, Gil-Guillen VF, Carratala-Munuera C (2019). Maternal periodontitis and preterm birth: systematic review and meta-analysis. Community Dent Oral Epidemiol.

[10] Peddis N, Musu D, Ideo F, Rossi-Fedele G, Cotti E (2019). Interaction of biologic therapy with apical periodontitis and periodontitis: a systematic review. Aust Dent J.

[11] Glanville JM, Lefebvre C, Miles JN, Camosso-Stefinovic J (2006). How to identify randomized controlled trials in MEDLINE: ten years on. J Med Libr Assoc.

[12] Durão S, Kredo T, Volmink J (2015). Validation of a search strategy to identify nutrition trials in PubMed using the relative recall method. J Clin Epidemiol.

[13] Golder S, Wright K, Loke YK (2018). The development of search filters for adverse effects of surgical interventions in medline and Embase. Health Inf Libr J.

[14] Budhram D, Navarro-Ruan T, Haynes RB (2018). The efficiency of database searches for creating systematic reviews was improved by search filters. J Clin Epidemiol.

[15] Lefebvre C, Glanville J, Beale S, Boachie C, Duffy S, Fraser C, Harbour J, McCool R, Smith L (2017). Assessing the performance of methodological search filters to improve the efficiency of evidence information retrieval: five literature reviews and a qualitative study. Health Technol Assess.

[16] Shariff SZ, Sontrop JM, Haynes RB, Iansavichus AV, McKibbon KA, Wilczynski NL, Weir MA, Speechley MR, Thind A, Garg AX (2012). Impact of PubMed search filters on the retrieval of evidence by physicians. CMAJ..

[17] Pillastrini P, Vanti C, Curti S, Mattioli S, Ferrari S, Violante FS, Guccione A (2015). Using PubMed search strings for efficient retrieval of manual therapy research literature. J Manip Physiol Ther.

[18] Bachmann LM, Coray R, Estermann P, Ter Riet G (2002). Identifying diagnostic studies in MEDLINE: reducing the number needed to read. J Am Med Inform Assoc.

[19] Olaussen A, Semple W, Oteir A, Todd P, Williams B (2017). Paramedic literature search filters: optimised for clinicians and academics. BMC Med Inform Decis Mak.

[20] Rollin L, Darmoni S, Caillard JF, Gehanno JF (2010). Searching for high-quality articles about intervention studies in occupational health--what is really missed when using only the Medline database?. Scand J Work Environ Health.

[21] Shea BJ, Reeves BC, Wells G (2017). AMSTAR 2: a critical appraisal tool for systematic reviews that include randomised or non-randomised studies of healthcare interventions, or both. BMJ..

[22] Spry C, Mierzwinski-Urban M (2018). The impact of the peer review of literature search strategies in support of rapid review reports. Res Synth Methods.

