# Supplementary material for: Validation of a search strategy for randomized clinical trials related to periodontitis
Source: Arch Public Health. 2021 Apr 3;79:43. doi: 10.1186/s13690-021-00560-0 (PMC8019511; doi:10.1186/s13690-021-00560-0)
Supplement: Supplementary file 1 — Additional file 1 Appendix A. Peer Review of Electronic Search Strategies (PRESS). Appendix B. PubMed search history (11/16/2018). [file 13690_2021_560_MOESM1_ESM.docx]

**APPENDIX A –** *Peer Review of Electronic Search Strategies* (PRESS)

***PRESS Guideline* — Search Submission & Peer Review Assessment**

**SEARCH SUBMISSION: THIS SECTION TO BE FILLED IN BY THE SEARCHER**

| **Searcher:** | Amanda Oliveira Lyrio | **Email:** | amandalyryo@hotmail.com |
| --- | --- | --- | --- |
| **Date submitted:** | 11/07/2018 | **Date requested by:** | 11/14/2018 |

**Systematic Review Title:**

Search strategy for randomized clinical trials related to periodontitis

**This search strategy is:**

| X | My PRIMARY (core) database strategy — First time submitting a strategy for search question and database |
| --- | --- |
|  | My PRIMARY (core) strategy — Follow-up review NOT the first time submitting a strategy for search question and database. If this is a response to peer review, itemize the changes made to the review suggestions |
|  | SECONDARY search strategy— First time submitting a strategy for search question and database |
|  | SECONDARY search strategy — NOT the first time submitting a strategy for search question and database. If this is a response to peer review, itemize the changes made to the review suggestions |

**Database**

(i.e., MEDLINE,CINAHL…):

Pubmed

**Interface**

(i.e., Ovid, EBSCO, PUBMED…):

Pubmed

**Research Question**

(Describe the purpose of the search)

What is the best search strategy for randomized clinical trials related to periodontitis?

**PI(E)COS Format**

(Outline the PICOs for your question — i.e., Patient, Index test, Reference standard, Outcome, and Study Design — as applicable)

| **P** | Human |
| --- | --- |
| **O** | Periodontitis |
| **S** | Randomized clinical trials |

**Inclusion Criteria**

(List criteria such as age groups, study designs, etc., to be included)

| **1** | Randomized clinical trials |
| --- | --- |
| **2** | Studies accompanying the diagnosis of confirmed periodontitis |

**Exclusion Criteria**

(List criteria such as study designs, date limits, etc., to be excluded)

| **1** | Animal studies |
| --- | --- |

**Was a search filter applied?**

Yes ( X )              	No (  )

**If YES, which one(s) (e.g., Cochrane RCT filter, PubMed Clinical Queries filter)? Provide the source if this is a published filter.** *[mandatory if YES to previous question]*

**(((((((((((randomized controlled trial[Publication Type]) OR controlled clinical trial[Publication Type]) OR controlled clinical trial[Title/Abstract]) OR placebo[Title/Abstract]) OR clinical trials as topic[MeSH Terms]) OR randomly[Title/Abstract]) OR trial[Title])) NOT ((animals [mh]) NOT humans [mh]))))**

**Other notes or comments you feel would be useful for the peer reviewer?**

**Please copy and paste your search strategy here, exactly as run, including the number of hits per line.** *[mandatory]*

|  | **((((((((((((((((Periodontitis[Title/Abstract]) OR Periodontitis[MeSH Terms]) OR Gingivitis[Title/Abstract]) OR Gingivitis[MeSH Terms]) OR Periodontal Diseases[Title/Abstract]) OR Periodontal Diseases[MeSH Terms]) OR Disease, Periodontal[Title/Abstract]) OR Diseases, Periodontal[Title/Abstract]) OR Periodontal Disease[Title/Abstract]) OR Parodontosis[Title/Abstract]) OR Parodontoses[Title/Abstract]) OR Pyorrhea Alveolaris[Title/Abstract]) OR Gingivitides[Title/Abstract]))))) AND ((((((((Cross-Sectional Studies[Mesh:noexp] OR cross-sectional[TIAB] OR Prevalence[mesh:noexp] OR prevalence[tiab] OR transversal study[tiab]))) OR ((“Case-Control Studies”[Mesh:noexp] OR "retrospective studies"[mesh:noexp] OR “Control Groups”[Mesh:noexp] OR (case[TIAB] AND control[TIAB]) OR (cases[TIAB] AND controls[TIAB]) OR (cases[TIAB] AND controlled[TIAB]) OR (case[TIAB] AND comparison*[TIAB]) OR (cases[TIAB] AND comparison*[TIAB]) OR “control group”[TIAB] OR “control groups”[TIAB]))) OR ((cohort studies[mesh:noexp] OR longitudinal studies[mesh:noexp] OR follow-up studies[mesh:noexp] OR prospective studies[mesh:noexp] OR retrospective studies[mesh:noexp] OR cohort[TIAB] OR longitudinal[TIAB] OR prospective[TIAB] OR retrospective[TIAB]))) OR “Epidemiologic Studies”[Mesh:noexp])) OR ((Incidence[mesh:noexp] OR incidence[tiab])))** | **25828** |
| --- | --- | --- |
| #1 | (((((((((((((((Periodontitis[Title/Abstract]) OR Periodontitis[MeSH Terms]) OR Gingivitis[Title/Abstract]) OR Gingivitis[MeSH Terms]) OR Periodontal Diseases[Title/Abstract]) OR Periodontal Diseases[MeSH Terms]) OR Disease, Periodontal[Title/Abstract]) OR Diseases, Periodontal[Title/Abstract]) OR Periodontal Disease[Title/Abstract]) OR Parodontosis[Title/Abstract]) OR Parodontoses[Title/Abstract]) OR Pyorrhea Alveolaris[Title/Abstract]) OR Gingivitides[Title/Abstract]))) | [96107](https://www.ncbi.nlm.nih.gov/pubmed/?cmd=HistorySearch&querykey=3) |
| #2 | (((((((((((randomized controlled trial[Publication Type]) OR controlled clinical trial[Publication Type]) OR controlled clinical trial[Title/Abstract]) OR placebo[Title/Abstract]) OR clinical trials as topic[MeSH Terms]) OR randomly[Title/Abstract]) OR trial[Title])) NOT ((animals [mh]) NOT humans [mh])))) | [1052178](https://www.ncbi.nlm.nih.gov/pubmed/?cmd=HistorySearch&querykey=50) |

**PEER REVIEW ASSESSMENT: THIS SECTION TO BE FILLED IN BY THE REVIEWER**

| **Reviewer:** Viviane Seixas Silva Silveira |  | **Email:** viviane.silveira@gmail.com |  |
| --- | --- | --- | --- |
| **Date completed:** 11/13/2018 | | | |

**1. TRANSLATION**

| A -­‐No revisions | **X** |
| --- | --- |
| B -­‐ Revision(s) suggested |  |
| C -­‐ Revision(s) required |  |

If “B” or “C,” please provide an explanation or example:

**2. BOOLEAN AND PROXIMITY OPERATORS**

| A -­‐No revisions | **X** |
| --- | --- |
| B -­‐ Revision(s) suggested |  |
| C -­‐ Revision(s) required |  |

If “B” or “C,” please provide an explanation or example:

**3. SUBJECT HEADINGS**

| A -­‐No revisions |  |
| --- | --- |
| B -­‐ Revision(s) suggested |  |
| C -­‐ Revision(s) required | **X** |

If “B” or “C,” please provide an explanation or example:

The terms “gingivitis” e “gingivitides” does not accurately translate the ending item of the acronym PICO, which is to evaluate the frequency of periodontitis, so I recommend removing them. In addition I recommend adding some synonyms of the term "periodontitis" in the title and abstract, for example: "periodontitides", "pericementitis", "pericementitides".

**4. TEXT WORD SEARCHING**

| A -­‐No revisions |  |
| --- | --- |
| B -­‐ Revision(s) suggested | **x** |
| C -­‐ Revision(s) required |  |

If “B” or “C,” please provide an explanation or example:

I recommend using the quotation marks, since the terms used are representative of PICO.

**5. SPELLING, SYNTAX, AND LINE NUMBERS**

| A -­‐No revisions | **x** |
| --- | --- |
| B -­‐ Revision(s) suggested |  |
| C -­‐ Revision(s) required |  |

If “B” or “C,” please provide an explanation or example:

**6. LIMITS AND FILTERS**

| A -­‐No revisions | **x** |
| --- | --- |
| B -­‐ Revision(s) suggested |  |
| C -­‐ Revision(s) required |  |

If “B” or “C,” please provide an explanation or example:

**OVERALL EVALUATION**

(Note:  If one or more “revision required” is noted above, the response below must be “revisions required”.)

| A -­‐No revisions |  |
| --- | --- |
| B -­‐ Revision(s) suggested |  |
| C -­‐ Revision(s) required | **x** |

Additional comments:

There are revisions that need to be performed, as described in the previous items.

**APPENDIX B -** PubMed search history (11/16/2018)

| Search | Add to builder | Query | Items found | Time |
| --- | --- | --- | --- | --- |
| #3 | Add | Search **((((((((((“Periodontitis” [Title/Abstract] OR “Periodontitis” [MeSH Terms] OR “Disease, Periodontal” [Title/Abstract] OR “Disease, Periodontal” [MeSH Terms] OR “Diseases, Periodontal” [Title/Abstract] OR “Periodontal Disease” [Title/Abstract] OR “Parodontosis” [Title/Abstract] OR “Parodontoses” [Title/Abstract] OR “Pyorrhea Alveolaris” [Title/Abstract])))) AND ( "2018/01/01"[PDat] : "2018/03/31"[PDat] ))) AND ((((((((((((((randomized controlled trial[Publication Type]) OR controlled clinical trial[Publication Type]) OR controlled clinical trial[Title/Abstract]) OR placebo[Title/Abstract]) OR clinical trials as topic[MeSH Terms]) OR randomly[Title/Abstract]) OR trial[Title])) NOT ((animals [mh]) NOT humans [mh])))))) AND ( "2018/01/01"[PDat] : "2018/03/31"[PDat] ))** Filters: **Publication date from 2018/01/01 to 2018/03/31** | [7](https://www.ncbi.nlm.nih.gov/pubmed/?cmd=HistorySearch&querykey=3)2 | 20:55:30 |
| #2 | Add | Search **(((((((“Periodontitis” [Title/Abstract] OR “Periodontitis” [MeSH Terms] OR “Disease, Periodontal” [Title/Abstract] OR “Disease, Periodontal” [MeSH Terms] OR “Diseases, Periodontal” [Title/Abstract] OR “Periodontal Disease” [Title/Abstract] OR “Parodontosis” [Title/Abstract] OR “Parodontoses” [Title/Abstract] OR “Pyorrhea Alveolaris” [Title/Abstract])))** Filters: **Publication date from 2018/01/01 to 2018/03/31** | 843 | 20:55:21 |
| #1 | Add | Search **((((((((((((randomized controlled trial[Publication Type]) OR controlled clinical trial[Publication Type]) OR** **randomized** **[Title/Abstract]) OR placebo[Title/Abstract]) OR clinical trials as topic[MeSH Terms]) OR randomly[Title/Abstract]) OR trial[Title])) NOT ((animals [mh]) NOT humans [mh])))))** Filters: **Publication date from 2018/01/01 to 2018/03/31** | 18056 | 20:54:47 |
